# Basket Trials: Past, Present, and Future

**DOI:** 10.1146/annurev-cancerbio-061421-012927

**Published:** 2023-12-05

**Authors:** Yonina R. Murciano-Goroff, Manik Uppal, Monica Chen, Guilherme Harada, Alison M. Schram

**Affiliations:** 1 Department of Medicine, Memorial Sloan Kettering Cancer Center, New York, NY, USA;; 2 Weill Cornell Medical College, New York, NY, USA

**Keywords:** basket trials, precision oncology, molecular oncology, genomic biomarkers, biomarker-selected trials, disease-agnostic drug development

## Abstract

Large-scale tumor molecular profiling has revealed that diverse cancer histologies are driven by common pathways with unifying biomarkers that can be exploited therapeutically. Disease-agnostic basket trials have been increasingly utilized to test biomarker-driven therapies across cancer types. These trials have led to drug approvals and improved the lives of patients while simultaneously advancing our understanding of cancer biology. This review focuses on the practicalities of implementing basket trials, with an emphasis on molecularly targeted trials. We examine the biologic subtleties of genomic biomarker and patient selection, discuss previous successes in drug development facilitated by basket trials, describe certain novel targets and drugs, and emphasize practical considerations for participant recruitment and study design. This review also highlights strategies for aiding patient access to basket trials. As basket trials become more common, steps to ensure equitable implementation of these studies will be critical for molecularly targeted drug development.

## INTRODUCTION

Cancer has traditionally been classified and treated on the basis of a tumor’s tissue of origin. Advances in genomic sequencing and large-scale efforts to molecularly profile tumors have identified recurrent genomic alterations across different cancer types, suggesting a unifying biology that may be susceptible to therapies targeting the aberrant pathway. As the number of potential targets has rapidly grown, there has been a shift toward a more disease-agnostic, biomarker-driven approach to drug development. Basket trials enroll patients on the basis of a specific biomarker regardless of tumor histology. Due to their efficiency in testing biomarker-driven hypotheses, and facilitated by the increased availability and quicker turnaround times of comprehensive molecular profiling, basket trials have rapidly become the study design of choice for early-phase targeted therapy trials (Adashek et al. 2023, Bedard et al. 2020, Cunanan et al. 2017, Donoghue et al. 2020, Haslam et al. 2023, Hyman et al. 2017b, Murciano-Goroff et al. 2020a, Tao et al. 2018, Tsimberidou et al. 2020, Wahida et al. 2023, West 2017).

**Figure 1:**
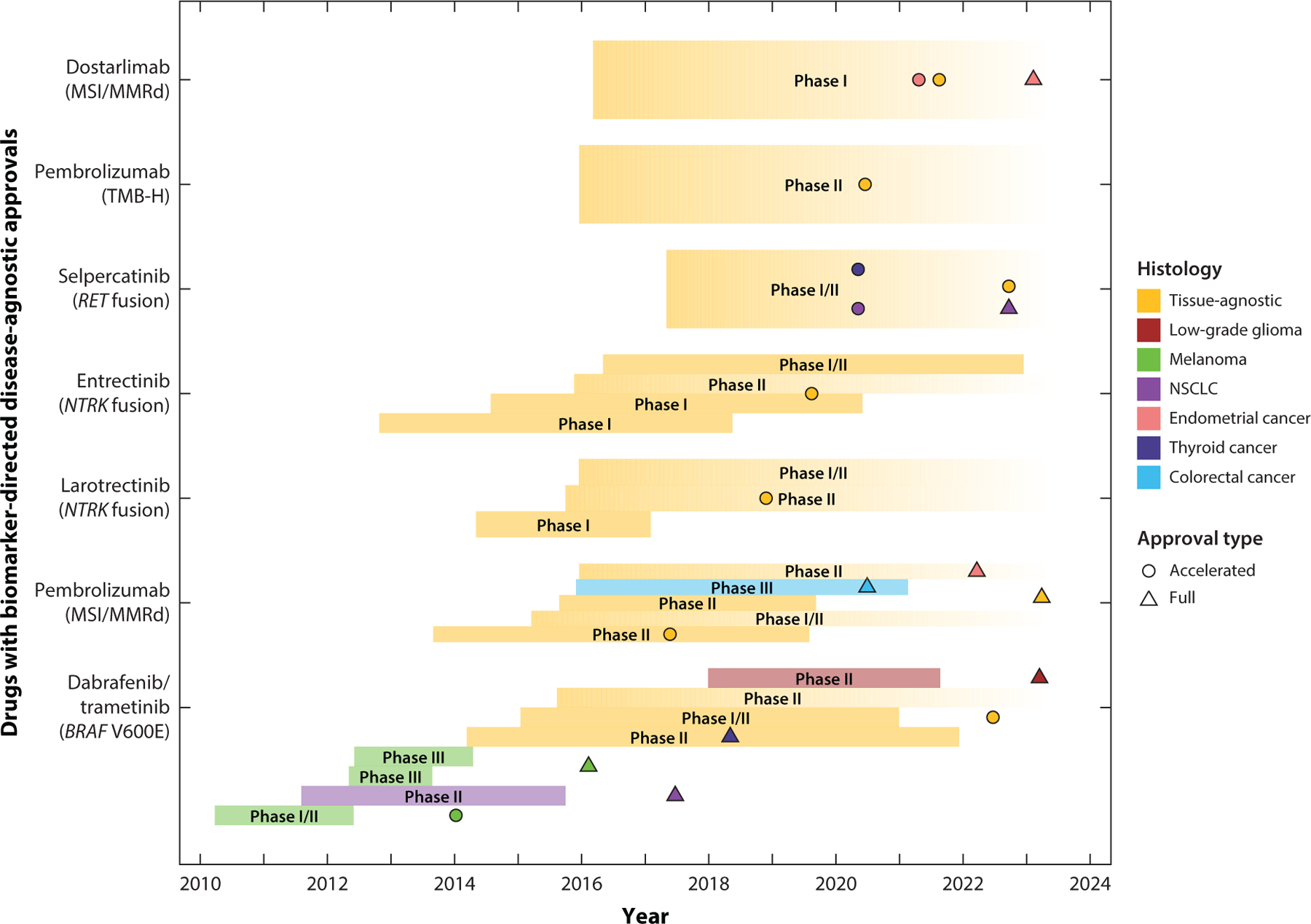
Timeline of clinical trials leading to disease-agnostic approvals by the US Food and Drug Administration. Abbreviations: MMRd, mismatch repair deficient; MSI, microsatellite instability; NSCLC, non-small-cell lung cancer; TMB-H, high tumor mutational burden.

Basket trials have led to many notable successes. In May 2017, the US Food and Drug Administration (FDA) issued its first tissue-agnostic regulatory approval: Pembrolizumab, a PD-1 inhibitor, was approved for adult and pediatric patients with unresectable or metastatic microsatellite instability–high (MSI-H) or mismatch repair–deficient (MMRd) solid tumors (André et al. 2020, Blumenthal & Pazdur 2018, Boyiadzis et al. 2018, Goldberg et al. 2018, Lemery et al. 2017). Soon thereafter, in November 2018, the FDA approved larotrectinib, a small-molecule inhibitor of TRK proteins, in adult or pediatric patients harboring *NTRK* fusions (Drilon 2019, Drilon et al. 2018). The pivotal studies leading to these landmark approvals were remarkable for their clear demonstration of efficacy regardless of tumor type. Several additional tumor-agnostic approvals for advanced solid tumors have followed (Duke et al. 2022, Gouda et al. 2023, Lavacchi et al. 2020, Moore & Guinigundo 2023, Tateo et al. 2023) (Figure 1).

Basket trials allow for interrogation of genomic alterations that are too rare to be practically studied in a disease-specific manner. Additionally, they provide a way to investigate rare tumor types for which independent trials would not be feasible (Tao et al. 2018). For example, *NTRK* fusions are enriched in infantile fibrosarcoma, secretory breast carcinoma, and mammary analog secretory carcinomas, diseases whose treatment has been revolutionized by the tissue-agnostic approval of larotrectinib (Siozopoulou et al. 2021).

The potential of basket trials lies not only in the opportunity for tumor-agnostic drug development but also in the wealth of knowledge generated by including multiple histologies and, in some cases, multiple biomarkers within the same trial (Murciano-Goroff et al. 2020a). The scientific insights gained from these studies improve our understanding of cancer biology, which can ultimately be exploited to develop more precise biomarkers of sensitivity and resistance to therapy, improve patient selection for trials, and create better drugs (Murciano-Goroff et al. 2020a, Rosen et al. 2022, Tao et al. 2018, Vasan et al. 2019). The prevalence of basket trials as a tool for accelerating drug development demands reflection on how the design of these trials can be optimized and tailored to address varied targets, heterogeneous drug classes, and diverse patient populations. In the remainder of this review, we discuss the practicalities of implementing basket trials based on tumor sequencing.

## OPTIMIZING BIOMARKER SELECTION

Improvements in genomic sequencing technologies have fueled the proliferation of basket trials. Prior to 2005, clinicians commonly used single-analyte tests relying on a polymerase chain reaction with Sanger sequencing to interrogate one mutation at a time. Consequently, tumors were typically tested only for biomarkers that had already been validated for a specific histology (Karlovich & Williams 2019). Over the past two decades, massive parallel DNA sequencing capable of evaluating multiple genes simultaneously has become widely available (Cronin & Ross 2011). This next-generation sequencing (NGS) approach continues to become faster, less expensive, more sensitive, and more accessible. Commercial NGS panels often contain more than 500 cancer-associated genes. These panels can detect mutations, insertions, deletions, copy number alterations, and some structural alterations (Morganti et al. 2019, Syn et al. 2016). Although not yet routinely used, whole-exome sequencing and whole-genome sequencing are becoming more widely available (Morganti et al. 2019). Improved detection of alterations has increased opportunities for therapeutic intervention.

In parallel, the number of therapies designed for specific genomic alterations in patients’ tumors has risen (Berger & Mardis 2018). Matching of patients to molecularly selected therapy has historically been considered dichotomous, with a tumor either having or not having the alteration of interest. However, the breadth of information provided by NGS testing has highlighted the molecular complexity of tumors, posing new challenges in addition to opportunities for identifying better biomarkers for novel treatments (Murciano-Goroff et al. 2020a).

**Figure 2:**
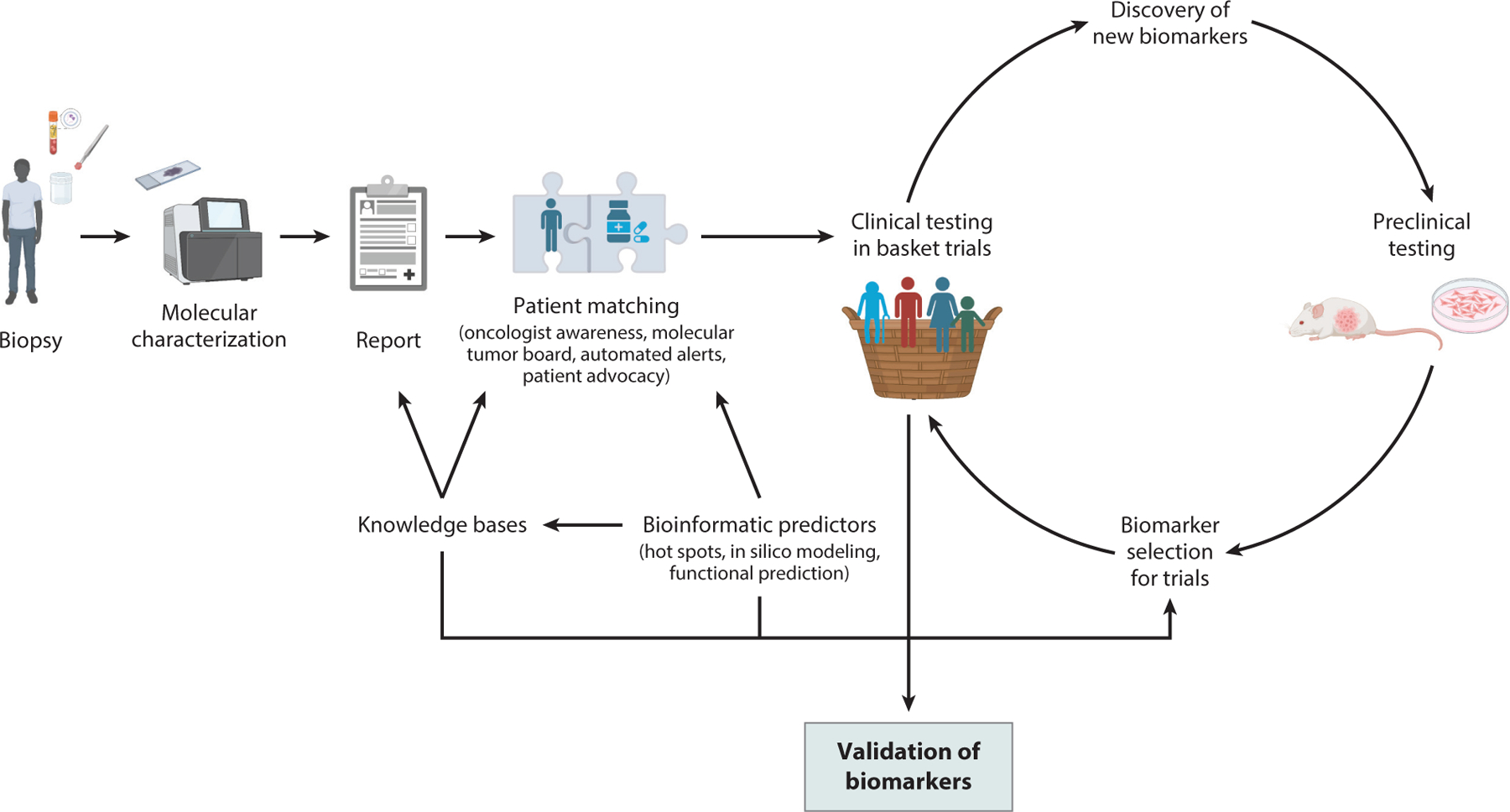
A schematic showing the relationships between patients, preclinical models, and basket trials that facilitate target validation. Better biomarkers lead to improved patient selection for clinical trials, and basket trials in turn contribute to the biological understanding of biomarkers that can drive future drug development. Figure adapted from images created with BioRender.com.

The key question when presented with an NGS report is whether the tumor contains a biomarker that may confer sensitivity to an approved or investigational therapy. Interpretation can be difficult, given the multiple variables that may influence tumor biology. Both histologic context and specific genetic/proteomic features (such as the unique amino acid change within a mutated gene, the level of amplification, or the coalterations observed) must be considered (Murciano-Goroff et al. 2020a). Ideally, genomic predictors of efficacy would be determined prior to designing trials, enabling enrichment for those subjects with the greatest chance of benefit. Practically, however, limitations on the scalability of preclinical modeling as well as the diversity of tumor histologies and molecular alterations have translated into bidirectional scientific investigations. As our understanding of cancer biology improves, we become better equipped to design rational biomarker-driven basket trials that enrich for those patients most likely to benefit. The results of clinical trials, in turn, advance biological knowledge, creating a positive cycle that has led to recent exponential growth in oncologic drug development (Bedard et al. 2020; Donoghue et al. 2020; Hyman et al. 2017b; Murciano-Goroff et al. 2020a,b) (Figure 2).

### Identifying Driver Alterations

Tumors typically contain numerous genomic alterations. Importantly, not all alterations have an equal role in tumorigenesis and tumor maintenance; there are different biologic consequences depending on the alteration (Chang et al. 2018, Li et al. 2023, Torkamani & Schork 2008). When identifying patients for a basket trial, it is important to consider whether the alteration being targeted is critical for the cancer’s survival. Preclinical modeling, including in vitro and in vivo studies, remains a useful tool for investigating whether a given alteration is oncogenic and likely to respond to a drug (Federici & Soddu 2020, Murciano-Goroff et al. 2020a). Unfortunately, it is not feasible to test every genomic alteration in the laboratory, and results may depend on the molecular and histologic context of the models used.

Computational analyses of large genomic data sets have identified recurrent mutations that occur in cancer more often than would be predicted by chance alone. These hot spots are often a consequence of positive selection, suggesting that they are important for tumor growth and/or survival and may be susceptible to targeted therapy (Chang et al. 2018). For example, this strategy identified small in-frame duplications in *AKT* as functionally relevant, and subsequent preclinical and clinical data confirmed that these alterations confer sensitivity to AKT inhibition (Shrestha Bhattarai et al. 2022). Other techniques to predict the biologic importance of variants of unknown significance (VUSes) include protein function prediction (such as using the PolyPhen-2 or SIFT tool) (Flanagan et al. 2010, Kerr et al. 2017), evolutionary conservation analysis (such as using GERP, CADD, or MutationAssessor) (Dong et al. 2015), saturation mutagenesis (Findlay et al. 2014), and multiplexed assays (Gasperini et al. 2016).

Notably, true driver oncogenes are often mutually exclusive in the treatment-naïve setting. In lung cancer, for example, de novo activating alterations in *EGFR*, *KRAS*, *ROS1*, *ALK*, *RET*, and *NTRK* rarely coexist (Farago & Azzoli 2017). The co-occurrence of *EGFR* and *KRAS* mutations negatively affects lung cancer development by inducing oncogenic stress and leading to synthetic lethality (Unni et al. 2015). The co-occurrence of a VUS alongside a known driver raises suspicion that the VUS is not itself a driver, though there are important exceptions, which may be disease dependent. In endometrial cancer, for example, multiple concurrent mutations involving *PIK3CA* and *PTEN* frequently coexist, amplifying PI3K/AKT pathway signaling (Sivakumar et al. 2023).

Specific features of a genomic alteration may also provide clues as to whether it is likely to be therapeutically relevant. Examples include the level of gene amplification (Lai et al. 2019), the size of the amplified region, zygosity, clonality, and whether the alteration is truncal (Donoghue et al. 2020). In general, a higher level of amplification may suggest a stronger selective pressure to overexpress a given protein and, therefore, a more appropriate genomic target. Additionally, when multiple genes are involved in a single amplification or deletion event, it can be difficult to know which the tumor was selecting for, as opposed to small amplified or deleted regions that are more likely to be oncogenic (Eifert & Powers 2012, Tanaka & Watanabe 2020).

Zygosity can also influence the likelihood of responding to targeted therapies. In tumor suppressors, biallelic alterations leading to loss of function may be required for therapeutic exploitation. Homozygous alterations, loss of heterozygosity favoring the mutant allele, or epi-genetic silencing of the wild-type allele may indicate functional losses with potential therapeutic relevance (Donoghue et al. 2020).

Truncality/clonality should also be accounted for when selecting therapeutic strategies. Truncal alterations develop early in tumorigenesis and are present across cancer cells. They are likely to be better targets than subclonal alterations in a limited number of cells. Although definitively determining clonality requires complex bioinformatic analysis, the presence of mutations with low mutant allele fractions in comparison to other alterations (accounting for zygosity) or the absence of the alteration in one or more good-quality tumor samples suggests that an alteration may not be clonal. Increasingly, multiregion sequencing and single-cell sequencing can map tumors’ clonal architecture. Although not yet widely available, this technology has the potential to identify patients with intratumoral heterogeneity who are less likely to respond to single-agent targeted therapy, thereby improving patient selection for clinical trials (Dentro et al. 2021, Lim et al. 2020, Liu et al. 2020).

### Allele-Specific Differences in Drug Sensitivity

Importantly, different mutations within the same gene may have different susceptibilities to targeted therapy. When these differences are known a priori, they can be accounted for in designing basket trials. In other cases, the trials themselves clarify such biological differences. For example, *BRAF* alterations are divided into three classes on the basis of how they activate cellular signaling. Currently approved BRAF inhibitors are effective in class I alterations that signal as constitutively active BRAF monomers, in contrast to class II and III alterations that signal as RAF-independent, high-kinase-activity dimers and RAF-dependent, low-kinase-activity dimers, respectively (Fontana & Valeri 2019, Yao et al. 2015). Newer RAF dimer inhibitors are being explored in class I–III *BRAF* alterations, although inhibition of upstream signaling may be required for effective targeting of class III alterations (Yao et al. 2017, 2019). Appreciation of such differences enables rational design of basket trial eligibility criteria.

### Histologic Context

The premise of basket trials is that tumors with the same genomic alterations may have shared, exploitable molecular dependencies. It is clear, however, that histology can influence response to targeted therapies. For example, colorectal cancers (CRCs) have demonstrated inferior efficacy compared with other histologies in several basket trials, including those testing BRAF and MEK inhibition (Prahallad et al. 2012, Salama et al. 2020), KRAS G12C inhibition (Fakih et al. 2022, Hong et al. 2020, Ou et al. 2022, Skoulidis et al. 2021), and even TRK inhibition (Drilon et al. 2018). Notably, the tumor-agnostic approval of dabrafenib (a BRAF inhibitor) in combination with trametinib (a MEK inhibitor) for *BRAF* V600E–mutant cancers explicitly excludes CRCs due to known intrinsic resistance (Gouda & Subbiah 2023). CRC’s poor sensitivity to some of these therapies is likely due to feedback activation of EGFR signaling; simultaneous inhibition of EGFR improves outcomes (Amodio et al. 2020, Kopetz et al. 2019, Yaeger et al. 2023).

The utility of poly(ADP-ribose) polymerase (PARP) inhibitors in *BRCA1*/*2*-altered cancers similarly illustrates the importance of histologic context. PARP inhibitors exploit loss-of-function *BRCA* mutations using synthetic lethality. Germline *BRCA1*/*2* mutations increase the inherited risk of developing BRCA-associated tumors, namely breast, ovarian, prostate, and pancreatic cancers. Despite the presence of *BRCA1*/*2* alterations across other histologies, the benefit of PARP inhibitors is limited primarily to BRCA-associated tumors, where this alteration appears to be the most biologically relevant (Jonsson et al. 2019, Murciano-Goroff et al. 2022, Schram et al. 2023a).

Histology also has a role in the frequency and mechanisms of acquired resistance. Gastrointestinal cancers harboring *TRK* fusions are more susceptible to off-target resistance to TRK inhibition mediated by activation of ERK signaling that may be overcome with MAPK pathway inhibition, while other tumor types are more likely to develop on-target *TRK* mutations that impair drug binding (Cocco et al. 2019).

Eligibility for basket trials must therefore account for available evidence regarding the histologies most likely to benefit, and histologies with predicted resistance may be excluded. For larger basket trials not limited by the rarity of the alteration of interest, individual disease-specific cohorts can be employed and analyzed both individually and in aggregate.

### Precision Medicine Knowledge Bases

Online databases have been developed to determine the predictive role of molecular alterations; these include OncoKB, OncoVAR, Jackson Laboratory Clinical Knowledgebase, CIViC, Precision Medicine Knowledgebase, MyCancerGenome, Cancer Genome Interpreter, CancerVar, MetaKB, and ClinVar. OncoKB was the first such database to be designated a public human genetic variant database by the FDA (2021), and it accounts for histologic context and specific alterations in more than 5,000 genes.

Different databases can have disparate interpretations of a variant’s oncogenicity (Wagner et al. 2020), and many alterations are still classified as VUSes. While basket trials with inclusive genomic eligibility criteria can help determine the clinical significance of VUSes, their inclusion increases the risk of enrolling patients with tumors not clearly driven by the biomarker of interest.

### Challenges in Biomarker Detection

While tissue-based DNA NGS panels that include numerous cancer-associated genes have been widely adopted, they do have limitations. They may detect gene fusions and alternative splice transcripts less reliably and are unable to identify changes in methylation or determine protein expression (Avila & Meric-Bernstam 2019, Di et al. 2019, Schram et al. 2017). RNA-based testing has emerged as a valuable tool to directly detect modified transcripts as well as to uncover targetable fusions and splice variants not detected by DNA testing (Benayed et al. 2019, Schram et al. 2017). This technique is particularly important for genes with large intronic regions that cannot practically be tiled into targeted DNA panels (such as *NRG1* fusions) (Nagasaka & Ou 2022). In non-small-cell lung cancer (NSCLC), for example, the integration of RNA and DNA sequencing can detect up to two to three times as many targetable fusions than DNA alone (Harada et al. 2023). An analysis of samples from the multihistology National Cancer Institute Molecular Analysis for Therapy Choice (NCI-MATCH) trial affirmed that many fusions, including actionable alterations with unusual breakpoints and/or unknown partners, may be missed by standard DNA assays (Kaluziak et al. 2023).

The routine incorporation of RNA-based testing into clinical care, while becoming more common, has been limited by the higher cost associated with multiple tests and the fact that RNA is more susceptible to degradation and preanalytical factors than DNA (Harada et al. 2023). Alternative methods also continue to be used. Break-apart fluorescence in situ hybridization is a fast and inexpensive way to detect fusions, but it can test only a limited number of genes simultaneously, cannot identify fusion partners, and can miss fusions with nearby partners or those that are out of frame (Schram et al. 2017). Immunohistochemistry (IHC) is often used to identify biomarkers for trials testing monoclonal antibodies or antibody–drug conjugates (ADCs), but more comprehensive proteomics is required to keep up with the growing list of trial biomarkers.

Tissue-based molecular profiling, regardless of the assay used, has the limitation of only testing a sample from a specific site of disease at a single time point, limiting information about spatial and temporal heterogeneity. Liquid biopsies analyzing tumor-derived cell-free DNA (cfDNA) from blood may enable a more comprehensive assessment of tumor heterogeneity and subclones, including resistance alterations, with a shorter turnaround time. Additionally, the ease of blood sampling allows for longitudinal tracking (Heitzer et al. 2019, Keller et al. 2021, Serrano et al. 2020). However, cfDNA poses new challenges for biomarker selection, including the incidental detection of clonal hematopoiesis (CH) and mosaicism. If plasma DNA sequencing alone is used, CH and mosaicism can lead to misinterpretation of mutations as tumor derived and might lead to trial enrollment on the basis of an alteration not present in the clinically relevant tumor (Heitzer et al. 2019, Köhnke & Majeti 2021, Serrano et al. 2020). In a study of patients who underwent paired solid tumor and blood sequencing, at least one CH mutation could be incorrectly attributed to the tumor in roughly 5% of patients (Ptashkin et al. 2018). Another study demonstrated that up to 10% of men with advanced prostate cancer have CH involving DNA repair genes, which have been used as biomarkers for trials in this population (Jensen et al. 2021). Patient-matched normal white blood cell DNA sequencing and bioinformatic algorithms can help distinguish tumor-derived genomic alterations from CH and mosaic variants (Brannon et al. 2021, Serrano et al. 2020).

## BASKET TRIALS: PAST, PRESENT, AND FUTURE

Many of the earliest basket trials involved disease-agnostic testing of drug–biomarker pairs that had previously been validated in a single histology in an attempt to expand the population of patients who benefit from targeted therapy. This testing was quickly followed by tumor-agnostic exploration of novel biomarkers. Select biomarkers are described below.

### HER2 Overexpression/Amplification and Mutations

HER2 overexpression is a validated biomarker in HER2^+^ breast and gastric cancer; however, its clinical relevance across tumors is poorly understood. The MyPathway study treated patients with HER2-overexpressed and/or -amplified nonbreast, nongastric cancers with trastuzumab and pertuzumab. Of the 199 *KRAS*-wild-type, efficacy-evaluable patients in this study, 26% had a confirmed response. Clinical activity differed between histologies, with an objective response rate (ORR) of 64% in salivary cancer; 25–33% in NSCLC, CRC, and biliary and pancreatic cancers; and below 17% in uterine, ovarian, and urothelial cancers (Meric-Bernstam et al. 2021). The national NCI-MATCH program ran a similar study (subprotocol J) of trastuzumab and pertuzumab in *HER2*-amplified solid tumors. The ORR was 8%, with one response each in colorectal cancer and cholangiocarcinoma (Connolly et al. 2020). A basket trial of the ADC ado-trastuzumab emtansine (T-DM1) similarly demonstrated disease-specific sensitivity, including efficacy in NSCLC and salivary gland carcinoma (Li et al. 2018). Another ADC, trastuzumab-deruxtecan, had an ORR of 61% in *HER2*^+^ (3+ by IHC) solid tumors (excluding breast, gastric, and lung cancers). The ORR was 44–85% in cervical, ovarian, endometrial, biliary tract, and bladder cancers, among others (Meric-Bernstam et al. 2023). The drug also has activity in *HER2*-mutant disease and has recently received approval for previously treated *HER2*-mutant NSCLC (Li et al. 2022b). *HER2* mutations have also been explored pan-cancer. The SUMMIT trial tested the pan–HER kinase inhibitor neratinib in *HER2*- and *HER3*-mutant tumors. Efficacy varied by both disease type and mutant allele; the greatest activity was observed in breast, cervical, and biliary cancers harboring kinase domain mutations (Hyman et al. 2018).

### MAPK Pathway Alterations

The MAPK pathway is commonly altered in cancer and has been the target of many basket trials, including with RAF inhibitors. Vemurafenib was studied in nonmelanoma *BRAF* V600–mutant tumors, and preliminary activity was identified in NSCLC, Erdheim–Chester disease, and Langerhans cell histiocytosis, among other cancers (Hyman et al. 2015). The international ROAR (Rare Oncology Agnostic Research) and NCI-MATCH subprotocol H trials investigated dabrafenib in combination with trametinib in *BRAF* V600E–mutant tumors. Activity was observed across disease types (Subbiah et al. 2020, 2022a, 2023a; Wen et al. 2022), leading to the FDA approval of this combination for solid tumors, excluding CRC (Salama et al. 2020). Several pan-RAF inhibitors and a BRAF-specific dimer breaker are under development for the treatment of *BRAF* non-V600 mutations with or without the addition of downstream MEK or ERK inhibition (Beck et al. 2023, De La Fuente et al. 2023, Schram et al. 2023b, Sullivan et al. 2020, Wang et al. 2023).

Other MAPK pathway targets have also been tested in basket trials. Initial dose escalation studies of the KRAS G12C inhibitors sotorasib and adagrasib were carried out pan-cancer, laying the groundwork for their accelerated approval in NSCLC (Hong et al. 2020, Ou et al. 2022). Basket trials are ongoing testing therapy for other *KRAS* alleles (https://www.clinicaltrials.gov identifiers NCT05379985, NCT05737706, NCT05533463, and NCT05382559, among others).

Inhibitors of downstream and upstream proteins in the MAPK pathway, including MEK, ERK, and SHP2, have been investigated using various MAPK alterations as biomarkers. The efficacy of these inhibitors as monotherapy has generally been limited (Brana et al. 2021, Eckstein et al. 2022, Sullivan et al. 2018), though notable exceptions include MEK inhibition for histiocytosis and low-grade serous ovarian cancer (LGSOC) (Diamond et al. 2019, Gershenson et al. 2022).

### PI3K Pathway Alterations

As with the MAPK pathway, several basket trials have targeted the PI3K-AKT pathway. For example, the PIK3CA inhibitor taselisib has been studied across 11 tumor types. While the response rate was limited, activity was observed in select histologies, including cervical as well as head and neck cancers (Jhaveri et al. 2021).

The AKT inhibitor capivasertib has been studied in *AKT1* E17K–mutant solid tumors, with responses in cervical, breast, lung, and endometrial cancers (Hyman et al. 2017a). The pan-AKT inhibitor TAS-117 was similarly tested in a basket trial of 13 patients with PI3K/AKT alterations. The ORR was 8%, with activity observed in patients with breast and ovarian cancers harboring select *PIK3CA* and *AKT* alterations (Lee et al. 2021). Downstream mTOR inhibition is being explored for patients with alterations in *TSC1*/*2* and/or other PI3K pathway alterations (Burris et al. 2022, Iyer et al. 2023).

### *FGFR* Alterations

Alterations in *FGFR1*–*3*, including fusions, amplifications, and mutations, have been observed across tumor types and have formed the basis of basket trials. The inhibitors pemigatinib, futibatinib, and RLY-4008 showed activity in multiple disease types and *FGFR* alterations (with RLY-4008 being *FGFR2*-specific), though additional follow-up is needed to determine the utility of targeting FGFR pan-cancer. Efficacy is most consistently demonstrated in *FGFR2* fusion–positive intrahepatic cholangiocarcinoma and *FGFR3*-mutant urothelial cancer (Borad et al. 2023, Meric-Bernstam et al. 2022, Rodon et al. 2023).

### Fusions

Oncogenic fusions have served as biomarkers for several notable basket trials. The TRK inhibitor larotrectinib received FDA approval for *TRK* fusion–positive cancers on the basis of pooled data from three basket trials. In an updated analysis, 244 adult and pediatric patients were efficacy evaluable across 25 different tumor types, most commonly soft tissue sarcoma and thyroid, lung, and salivary gland tumors. Patients had gene fusions involving *NTRK1* (46%), *NTRK2* (3%), or *NTRK3* (51%). The ORR was 69%, with clinically relevant activity observed across diseases and TRK isoforms (Drilon et al. 2022). A pooled analysis from three basket trials (ALKA, STARTRK-1, and STARTRK-2) testing another TRK inhibitor, entrectinib, in *TRK* fusion–positive cancers demonstrated similar results, with an ORR of 61% (92 of 150 patients) and 17 tumor types represented (Krzakowski et al. 2022).

The entrectinib basket trials also included patients with *ROS1* and *ALK* fusions. In the phase II eligible population, the ORR was 86% among 14 patients with *ROS1*-rearranged disease (all but one with NSCLC), and an ORR of 57% among seven patients with *ALK*-rearranged tumors, including NSCLC, renal cell carcinoma, and CRC (Drilon et al. 2017). A similar pediatric trial, the STARTRK-NG trial, enrolled 43 pediatric patients across tumors, with an ORR of 58% (Desai et al. 2022). The NCI-MATCH subprotocols F and G treated patients with *ALK*- or *ROS1-*rearranged tumors, respectively, with crizotinib. Two of four (50%) eligible *ALK* fusion–positive patients responded to treatment (one with leiomyosarcoma and the other with CRC). One of four patients with a *ROS1* fusion–positive tumor responded (24%), namely a patient with LGSOC (Mansfield et al. 2022).

The RET inhibitor selpercatinib was studied in the phase 1/2 LIBRETTO-001 basket trial for *RET* fusion–positive tumors. The ORR was 44%. Promising early activity was observed in *RET* fusion–positive NSCLC as well as in *RET* mutant– and *RET* fusion–positive thyroid cancer, leading to FDA approval of selpercatinib in these disease-specific indications followed by tumor-agnostic approval (Drilon et al. 2020, Subbiah et al. 2022b, Wirth et al. 2020).

### Microsatellite Instability and High Tumor Mutational Burden

Immunotherapies have received tissue-agnostic approvals based on basket trials. A deficiency in genes responsible for mismatch repair (MMRd) can lead to tumors with genomic microsatellite instability (MSI-H). MMRd/MSI-H tumors are especially susceptible to immune checkpoint blockade (André et al. 2020; Ganesh et al. 2019; Latham et al. 2019; Le et al. 2015, 2017), as are tumors with high tumor mutational burden (TMB), likely due to increased neoantigen production (Samstein et al. 2019). The KEYNOTE-158 trial built on the success of pembrolizumab for MMRd/MSI-H colorectal cancers by testing the drug in other histologies. A total of 27 different MMRd/MSI-H tumor types were represented among 233 patients, with an ORR of 34% (Marabelle et al. 2020b). In a larger analysis of 790 efficacy-evaluable patients for whom TMB testing was available, a 29% response rate was observed among the 102 patients with TMB of 10 or greater, in comparison to 6% among patients with lower TMB (Marabelle et al. 2020a).

### DNA Repair

The first reported basket trial enrolled patients with germline *BRCA1*/*2* mutations to the PARP inhibitor olaparib (Fong et al. 2009). *BRCA1*/*2* mutations were enriched in the dose escalation and required for the expansion. As noted above, objective responses were observed only in *BRCA1*/*2* mutation carriers with BRCA-associated cancers. The combination of PARP inhibition and immunotherapy was more recently explored in the JAVELIN BRCA/ATM study, which tested talazoparib along with avelumab, and the MEDIOLA trial, which tested olaparib in combination with durvalumab. These studies also demonstrated notable clinical activity predominantly in BRCA-associated tumor types (Domchek et al. 2020, Krebs et al. 2023, Schram et al. 2023a). Given the success of PARP inhibitors in *BRCA*-mutant tumors, drugs targeting other proteins important for DNA repair, such as ATR, WEE1, and PKMYT1, are being explored in basket trials using a range of biomarkers (e.g., NCT04855656, NCT04266912, NCT0318896, NCT04855656, NCT04768868).

### Novel Targets

Recent basket trials have addressed an expanded range of biologic vulnerabilities of cancer. A growing number of previously undruggable targets have become targetable through advances in chemistry, computer modeling, dynamic simulation, and drug development. KRAS allele–specific inhibitors and multi-inhibitors (Hofmann et al. 2022, Hong et al. 2020, Kemp et al. 2023, Kim et al. 2023, Lito et al. 2016, Ou et al. 2022), p53 Y220C inhibitors (Dumbrava et al. 2022), isoform-selective FGFR inhibitors (Subbiah et al. 2023b), and mutant-specific PIK3CA inhibitors (Perez et al. 2022) all aim to overcome previous drug development challenges. In addition to an expanding list of genetic targets, a growing number of overexpressed surface proteins are being used to direct novel therapies, such as ADCs and bispecific antibodies, to cancer cells (Drago et al. 2021, Meric-Bernstam et al. 2018). Detection of immunologic markers on cancer cells, such as expression of checkpoint proteins or neoantigens (whether a specific neoantigen or assumed neoantigen excess in MSI-H or high-TMB tumors), has enabled basket trials of immune therapies, as discussed above (Friedman et al. 2022, Murciano-Goroff et al. 2020b, Patel et al. 2020, Tateo et al. 2023).

The molecular biomarker for a basket trial no longer needs to be the drug’s direct target. Increasingly, basket trials are designed by utilizing knowledge of pathway signaling and synthetic lethality to target the functional consequences of molecular alterations. Examples of attempts to target downstream of the biomarker of interest, which have met with variable success, have included basket trials testing pan-RAF inhibitors and/or MEK and ERK inhibition in *RAS-*mutant cancers (Chenard-Poirier et al. 2017; Sullivan et al. 2018, 2020); MEK inhibition in *NF1*-, *GNAQ*-, and *GNA11*-mutant tumors (Wisinski et al. 2023); and mTOR inhibitors for the treatment of *TSC1*/*2*-, *STK11*-, *PTEN*-, and/or *PIK3CA*-mutant tumors (NCT02465060, NCT04774952). *NRG1* fusions are oncogenic ligands that signal through binding to the *HER3* receptor. Antibodies targeting HER3 are being explored in tumor-agnostic studies, with promising efficacy reported thus far (Carrizosa et al. 2022, Schram et al. 2022). Similarly, *PTCH1* loss-of-function mutations relieve inhibition of SMO, and SMO inhibitors are being investigated in basket trials of *PTCH1*-altered tumors (NCT02465060). Synthetic lethal approaches being investigated in tumor-agnostic studies include PARP inhibitors for *BRCA1*/*2*- or *ATM*-mutant cancers (Schram et al. 2023a); ATR inhibitors for *ATM*-mutant cancers (Yap et al. 2021); and PKMYT1 inhibition for solid tumors with *CCNE1* amplification, *FBXW7* loss, and *PPP2R1A* mutations (NCT04855656).

### Novel Therapeutics

To target the growing list of potential biologic vulnerabilities, a broader range of drug classes are being explored beyond traditional small molecules. Basket trials are being designed to test monoclonal and bispecific antibodies, ADCs, proteolysis-targeting chimeras, T cell receptors, and vaccines (Murciano-Goroff et al. 2020a,b).

## FACILITATING ENROLLMENT IN BASKET TRIALS

Basket trials entail unique challenges that compound already profound systemic barriers in access to clinical trials. Difficulty identifying appropriate studies, geographic barriers, financial costs, narrow eligibility criteria, attitudes regarding trials, and knowledge of trial availability all limit trial enrollment (Esdaille et al. 2022, Lara et al. 2005, Mohd Noor et al. 2013, Sharrocks et al. 2014, Unger et al. 2021). Fewer than 5% of adult patients with cancer enroll in studies, despite 70% being willing to participate (Comis et al. 2003, Lara et al. 2005, Lynam et al. 2012, Murthy et al. 2004, Tejeda et al. 1996). Enrollment in molecularly selected basket trials has the added barrier of requiring genomic sequencing (which may not be covered by insurance) and appropriate trial matching (Arora et al. 2022, Liu et al. 2022, McCarthy et al. 2016). Additionally, trials for rare genetic alterations may not be available at all centers, creating travel-related financial burdens. Unsurprisingly, patients required to travel more than 120 miles are less likely to enroll in a trial (Uehara et al. 2023).

Recently, the FDA (2022) released a draft guidance outlining measures to facilitate the enrollment of underserved populations through community engagement, language assistance, and reduction of trial burdens (such as allowing the use of local laboratory testing to minimize patient travel). As telemedicine has gained acceptance in the era of COVID-19, many have questioned whether remote care and local monitoring can be further incorporated into trials (Li et al. 2022a). Increased uptake of tumor molecular profiling, expansion of the oncogenic targets being explored, better mechanisms for patient matching, and improved access to trials can maximize equity and efficiency in enrollment. Novel trial designs, such as multicenter master protocols and just-in-time trials, may further facilitate basket trials enrollment.

### Next-Generation Sequencing Reports

NGS reports from commercial vendors such as Foundation Medicine, Caris, and Tempus include a nonexhaustive list of potential clinical trials (Simon & Roychowdhury 2013). Some trial sponsors have partnered with genomic sequencing companies to enable direct alerts to providers, though companies have limited clinical information with which to evaluate patient eligibility and dynamic changes in trial availability.

### Clinical Trial Matching Services

Some academic centers have created automated trial matching systems. The DARWIN Cohort Management System at Memorial Sloan Kettering Cancer Center sends automated notifications to physicians when patients have been identified who may be eligible for a clinical trial on the basis of a molecular feature (Tao et al. 2019). This matching system is facilitated by a large internal genomic sequencing effort, limiting its generalizability. Similarly, MatchMiner is an open-source platform developed at the Dana-Farber Cancer Institute with the goal of accelerating patient–trial matching (Klein et al. 2022). Several private companies, such as Caris and Foundation Medicine, as well as nonprofit organizations, are developing trial matching services wherein patients provide detailed clinical information, either through engagement with trained trial navigators or through an online interface (see https://www.carislifesciences.com/products-and-services/clinical-trials, https://www.foundationmedicine.com/service/clinical-research-and-trial-matching).

### Bringing Trials to the Patient

Another potential solution for facilitating greater trial enrollment is to allow community oncology practices to become on-demand clinical trial sites. In just-in-time trials, local sites undergo activation only when a patient has been identified, eliminating the need for start-up at nonen-rolling centers and allowing smaller sites to provide trial access (Lynam et al. 2012, Wiener et al. 2007). Numerous companies have assisted in this model. For example, TEMPUS has released data showing an average time to activation of 9.4 business days for its trials, far shorter than the estimated baseline average of 20-plus weeks, with a mean time from activation to patient consent of 4.5 days (Oleary et al. 2021). Similarly, the Caris Right-in-Time Clinical Trials program aims for 3 to 12 days to site activation, with patients enrolled by day 14 (see https://www.carislifesciences.com/products-and-services/clinical-trials/right-in-time).

### Multicenter Master Protocols

The creation of multicenter, multiarm studies has improved access to basket trials. These studies enroll patients into one of several treatment arms matched to their particular alteration, enabling access to multiple therapies across different trial sites. These master protocols have demonstrated the feasibility of enrolling thousands of patients. The NCI-MATCH and pediatric MATCH trials assigned patients in a disease-agnostic fashion to treatment arms on the basis of molecular alterations (Murciano-Goroff et al. 2021). More than 6,391 patients were enrolled in the adult NCI-MATCH trial, with 18% ultimately assigned to a treatment arm following tumor procurement and sequencing. Response rates were variable, ranging from no responses in certain arms to 38% in the dabrafenib/trametinib combination arm for *BRAF* V600E/K–mutant tumors (Murciano-Goroff et al. 2021). The MyPathway and Targeted Agent and Profiling Utilization Registry (TAPUR) trials followed a similar paradigm across countless study sites (Hainsworth et al. 2018, Klute et al. 2022, Mangat et al. 2018, Meric-Bernstam et al. 2019).

### Expanded-Access and Single-Patient Protocols

Expanded-access and single-patient protocols provide investigational therapy to patients who cannot enroll in a registrational trial as a result of exclusionary clinical factors or practical barriers (Feit et al. 2019, Scepura et al. 2021). The patient’s oncologist is provided with or creates a treatment plan that undergoes institutional review board approval. Efforts to facilitate improved data capture from these patient-centered trials are underway (Polak et al. 2023).

## SPECIAL CONSIDERATIONS IN STUDY DESIGN

### Statistical Considerations

Basket trials pose unique statistical challenges due to the inclusion of heterogeneous diseases with differing bars for clinically relevant efficacy. While a full exploration of basket trial statistics is beyond the scope of this review (Kaizer et al. 2019), it is notable that randomization is rarely feasible given differing standards of care. The majority of basket trials have been exploratory, signal-seeking phase I or II studies that pool results from all histologies and/or report disease-specific efficacy. Alternative two-stage designs have been proposed wherein the inactive indications are pruned at an interim analysis and the active indications are pooled in the final analysis. Given the inflated error potential with cherry-picking, the type 1 error rate needs to be controlled (Chen et al. 2016, Zhou et al. 2019).

### Regulatory Considerations

A critical question is what level of evidence justifies disease-agnostic approval versus further testing in individual histologies. Both the US FDA and the European Medicines Agency have offered guidance emphasizing the need for a strong scientific and clinical rationale before considering disease-agnostic approvals (EMA 2021, US Dep. Health Hum. Serv. et al. 2022). More explicit international criteria are likely to emerge, given the growing number of pan-cancer basket trials.

## CONCLUSION

Basket trials have become increasingly common in oncology and can be used to achieve tumor-agnostic drug approvals or explore biologic differences in disparate tumor types unified by a common molecular alteration. Several successful genome-directed therapies have stemmed from this study design, providing additional therapeutic options to patients with tumors harboring specific genomic alterations. As the field moves forward, it is critical to optimize biomarker and patient selection, expand molecular testing, streamline clinical trial matching, and improve access to basket studies.

## References

[R1] Adashek JJ, Subbiah V, Westphalen CB, Naing A, Kato S, Kurzrock R. 2023. Cancer: slaying the nine-headed Hydra. Ann. Oncol 34:61–69 35931318 10.1016/j.annonc.2022.07.010 PMC10923524

[R2] Amodio V, Yaeger R, Arcella P, Cancelliere C, Lamba S, 2020. EGFR blockade reverts resistance to *KRAS*^*G12C*^ inhibition in colorectal cancer. Cancer Discov. 10:1129–39 32430388 10.1158/2159-8290.CD-20-0187 PMC7416460

[R3] André T, Shiu KK, Kim TW, Jensen BV, Jensen LH, 2020. Pembrolizumab in microsatellite-instability-high advanced colorectal cancer. N. Engl. J. Med 383:2207–18 33264544 10.1056/NEJMoa2017699

[R4] Arora K, Tran TN, Kemel Y, Mehine M, Liu YL, 2022. Genetic ancestry correlates with somatic differences in a real-world clinical cancer sequencing cohort. Cancer Discov. 12:2552–65 36048199 10.1158/2159-8290.CD-22-0312 PMC9633436

[R5] Avila M, Meric-Bernstam F. 2019. Next-generation sequencing for the general cancer patient. Clin. Adv. Hematol. Oncol 17:447–54 31449513 PMC6739831

[R6] Beck JTT, McKean M, Gadgeel SM, Bowles DW, Haq R, 2023. A phase 1, open-label, dose escalation and dose expansion study to evaluate the safety, tolerability, pharmacokinetics, and antitumor activity of PF-07799933 (ARRY-440) as a single agent and in combination therapy in participants 16 years and older with advanced solid tumors with BRAF alterations. J. Clin. Oncol 41:TPS3164

[R7] Bedard PL, Hyman DM, Davids MS, Siu LL. 2020. Small molecules, big impact: 20 years of targeted therapy in oncology. Lancet 395:1078–88 32222192 10.1016/S0140-6736(20)30164-1

[R8] Benayed R, Offin M, Mullaney K, Sukhadia P, Rios K, 2019. High yield of RNA sequencing for targetable kinase fusions in lung adenocarcinomas with no mitogenic driver alteration detected by DNA sequencing and low tumor mutation burden. Clin. Cancer Res 25:4712–22 31028088 10.1158/1078-0432.CCR-19-0225 PMC6679790

[R9] Berger MF, Mardis ER. 2018. The emerging clinical relevance of genomics in cancer medicine. Nat. Rev. Clin. Oncol 15:353–65 29599476 10.1038/s41571-018-0002-6 PMC6658089

[R10] Blumenthal GM, Pazdur R. 2018. Approvals in 2017: gene therapies and site-agnostic indications. Nat. Rev. Clin. Oncol 15:127–28 29384145 10.1038/nrclinonc.2018.11

[R11] Borad MJ, Schram AM, Kim RD, Kamath SD, Sahai V, 2023. Updated dose escalation results for Re-Focus, a first-in-human study of highly selective *FGFR2* inhibitor RLY-4008 in cholangiocarcinoma and other solid tumors. J. Clin. Oncol 41:4009

[R12] Boyiadzis MM, Kirkwood JM, Marshall JL, Pritchard CC, Azad NS, Gulley JL. 2018. Significance and implications of FDA approval of pembrolizumab for biomarker-defined disease. J. Immunother. Cancer 6:35 29754585 10.1186/s40425-018-0342-x PMC5950135

[R13] Brana I, Shapiro G, Johnson ML, Yu HA, Robbrecht D, 2021. Initial results from a dose finding study of TNO155, a SHP2 inhibitor, in adults with advanced solid tumors. J. Clin. Oncol 39:3005

[R14] Brannon AR, Jayakumaran G, Diosdado M, Patel J, Razumova A, 2021. Enhanced specificity of clinical high-sensitivity tumor mutation profiling in cell-free DNA via paired normal sequencing using MSK-ACCESS. Nat. Commun 12:3770 34145282 10.1038/s41467-021-24109-5 PMC8213710

[R15] Burris HA III, Ulahannan SV, Haura EB, Ou S-HI, Capasso A, 2022. The bi-steric mTORC1-selective inhibitor RMC-5552 in tumors with activation of mTOR signaling: Preclinical activity in combination with RAS(ON) inhibitors in RAS-addicted tumors, and initial clinical findings from a single agent phase 1/1b study. J. Clin. Oncol 40:3098–9836070625

[R16] Carrizosa DR, Burkard ME, Elamin YY, Desai J, Gadgeel SM, 2022. CRESTONE: initial efficacy and safety of seribantumab in solid tumors harboring *NRG1* fusions. J. Clin. Oncol 40:300635786967

[R17] Chang MT, Bhattarai TS, Schram AM, Bielski CM, Donoghue MTA, 2018. Accelerating discovery of functional mutant alleles in cancer. Cancer Discov. 8:174–83 29247016 10.1158/2159-8290.CD-17-0321 PMC5809279

[R18] Chen C, Li X, Yuan S, Antonijevic Z, Kalamegham R, Beckman RA. 2016. Statistical design and considerations of a phase 3 basket trial for simultaneous investigation of multiple tumor types in one study. Stat. Biopharm. Res 8:248–57

[R19] Chenard-Poirier M, Kaiser M, Boyd K, Sriskandarajah P, Constantinidou A, 2017. Results from the biomarker-driven basket trial of RO5126766 (CH5127566), a potent RAF/MEK inhibitor, in *RAS*- or *RAF*-mutated malignancies including multiple myeloma. J. Clin. Oncol 35(15 Suppl.):2506

[R20] Cocco E, Schram AM, Kulick A, Misale S, Won HH, 2019. Resistance to TRK inhibition mediated by convergent MAPK pathway activation. Nat. Med 25:1422–27 31406350 10.1038/s41591-019-0542-z PMC6736691

[R21] Comis RL, Miller JD, Aldigé CR, Krebs L, Stoval E. 2003. Public attitudes toward participation in cancer clinical trials. J. Clin. Oncol 21:830–35 12610181 10.1200/JCO.2003.02.105

[R22] Connolly RM, Wang V, Hyman DM, Grivas P, Mitchell E, 2020. 553P activity of trastuzumab and pertuzumab (HP) in patients with non-breast/gastroesophgeal HER2-amplified tumours: results of the NCI-MATCH trial (EAY131) subprotocol J. Ann. Oncol 31(Suppl. 4):479–80

[R23] Cronin M, Ross JS. 2011. Comprehensive next-generation cancer genome sequencing in the era of targeted therapy and personalized oncology. Biomark. Med 5:293–305 21657839 10.2217/bmm.11.37

[R24] Cunanan KM, Gonen M, Shen R, Hyman DM, Riely GJ, 2017. Basket trials in oncology: a trade-off between complexity and efficiency. J. Clin. Oncol 35:271–73 27893325 10.1200/JCO.2016.69.9751 PMC5559900

[R25] De La Fuente MI, Rodon Ahnert J, Yaeger R, Tsai FY-C, Janku F, 2023. Safety and efficacy of the novel *BRAF* inhibitor FORE8394 in patients with advanced solid and CNS tumors: results from a phase 1/2A study. J. Clin. Oncol 41:3006

[R26] Dentro SC, Leshchiner I, Haase K, Tarabichi M, Wintersinger J, 2021. Characterizing genetic intra-tumor heterogeneity across 2,658 human cancer genomes. Cell 184:2239–54.e39 33831375 10.1016/j.cell.2021.03.009 PMC8054914

[R27] Desai AV, Robinson GW, Gauvain K, Basu EM, Macy ME, 2022. Entrectinib in children and young adults with solid or primary CNS tumors harboring *NTRK*, *ROS1*, or *ALK* aberrations (STARTRK-NG). Neuro Oncol. 24:1776–89 35395680 10.1093/neuonc/noac087 PMC9527518

[R28] Di C, Syafrizayanti Zhang Q, Chen Y, Wang Y, 2019. Function, clinical application, and strategies of pre-mRNA splicing in cancer. Cell Death Differ. 26:1181–94 30464224 10.1038/s41418-018-0231-3 PMC6748147

[R29] Diamond EL, Durham BH, Ulaner GA, Drill E, Buthorn J, 2019. Efficacy of MEK inhibition in patients with histiocytic neoplasms. Nature 567:521–24 30867592 10.1038/s41586-019-1012-y PMC6438729

[R30] Domchek SM, Postel-Vinay S, Im SA, Park YH, Delord JP, 2020. Olaparib and durvalumab in patients with germline *BRCA*-mutated metastatic breast cancer (MEDIOLA): an open-label, multicentre, phase 1/2, basket study. Lancet Oncol. 21:1155–64 32771088 10.1016/S1470-2045(20)30324-7

[R31] Dong C, Wei P, Jian X, Gibbs R, Boerwinkle E, 2015. Comparison and integration of deleteriousness prediction methods for nonsynonymous SNVs in whole exome sequencing studies. Hum. Mol. Genet 24:2125–37 25552646 10.1093/hmg/ddu733 PMC4375422

[R32] Donoghue MTA, Schram AM, Hyman DM, Taylor BS. 2020. Discovery through clinical sequencing in oncology. Nat. Cancer 1:774–83 35122052 10.1038/s43018-020-0100-0 PMC8985175

[R33] Drago JZ, Modi S, Chandarlapaty S. 2021. Unlocking the potential of antibody–drug conjugates for cancer therapy. Nat. Rev. Clin. Oncol 18:327–44 33558752 10.1038/s41571-021-00470-8 PMC8287784

[R34] Drilon A 2019. TRK inhibitors in TRK fusion–positive cancers. Ann. Oncol 30(Suppl. 8):23–30 10.1093/annonc/mdz282 32223935

[R35] Drilon A, Laetsch TW, Kummar S, DuBois SG, Lassen UN, 2018. Efficacy of larotrectinib in TRK fusion–positive cancers in adults and children. N. Engl. J. Med 378:731–39 29466156 10.1056/NEJMoa1714448 PMC5857389

[R36] Drilon A, Oxnard GR, Tan DSW, Loong HHF, Johnson M, 2020. Efficacy of selpercatinib in RET fusion–positive non-small-cell lung cancer. N. Engl. J. Med 383:813–24 32846060 10.1056/NEJMoa2005653 PMC7506467

[R37] Drilon A, Siena S, Ou S-HI, Patel M, Ahn MJ, 2017. Safety and antitumor activity of the multitargeted pan-TRK, ROS1, and ALK inhibitor entrectinib: combined results from two phase I trials (ALKA-372-001 and STARTRK-1). Cancer Discov. 7:400–9 28183697 10.1158/2159-8290.CD-16-1237 PMC5380583

[R38] Drilon AE, Hong DS, van Tilburg CM, Doz F, Tan DSW, 2022. Long-term efficacy and safety of larotrectinib in a pooled analysis of patients with tropomyosin receptor kinase (TRK) fusion cancer. J. Clin. Oncol 40:3100

[R39] Duke ES, Fusco MJ, DeMoss P, Dilawari A, Pamuk GE, 2022. Highlights of FDA oncology approvals in 2022: tissue-agnostic indications, dosage optimization, and diversity in drug development. Cancer Discov. 12:2739–46 36458428 10.1158/2159-8290.CD-22-1185

[R40] Dumbrava EE, Johnson ML, Tolcher AW, Shapiro G, Thompson JA, 2022. First-in-human study of PC14586, a small molecule structural corrector of Y220C mutant p53, in patients with advanced solid tumors harboring a *TP53 Y220C* mutation. J. Clin. Oncol 40:300335594490

[R41] Eckstein OS, Allen CE, Williams PM, Roy-Chowdhuri S, Patton DR, 2022. Phase II study of selumetinib in children and young adults with tumors harboring activating mitogen-activated protein kinase pathway genetic alterations: arm E of the NCI-COG pediatric MATCH trial. J. Clin. Oncol 40:2235–45 35363510 10.1200/JCO.21.02840 PMC9273373

[R42] Eifert C, Powers RS. 2012. From cancer genomes to oncogenic drivers, tumour dependencies and therapeutic targets. Nat. Rev. Cancer 12:572–78 22739505 10.1038/nrc3299

[R43] EMA (Eur. Med. Agency). 2021. Guideline on the clinical evaluation of anticancer medicinal products. EMA, Amsterdam. https://www.ema.europa.eu/en/documents/scientific-guideline/draft-guideline-evaluation-anticancer-medicinal-products-man-revision-6_en.pdf

[R44] Esdaille AR, Ibilibor C, Holmes A 2nd, Palmer NR, Murphy AB. 2022. Access and representation: a narrative review of the disparities in access to clinical trials and precision oncology in Black men with prostate cancer. Urology 163:90–98 34582887 10.1016/j.urology.2021.09.004

[R45] Fakih MG, Kopetz S, Kuboki Y, Kim TW, Munster PN, 2022. Sotorasib for previously treated colorectal cancers with *KRAS^G12C^* mutation (CodeBreaK100): a prespecified analysis of a single-arm, phase 2 trial. Lancet Oncol. 23:115–24 34919824 10.1016/S1470-2045(21)00605-7

[R46] Farago AF, Azzoli CG. 2017. Beyond *ALK* and *ROS1*: *RET*, *NTRK*, *EGFR* and *BRAF* gene rearrangements in non-small-cell lung cancer. Transl. Lung Cancer Res 6:550–59 29114471 10.21037/tlcr.2017.08.02 PMC5653525

[R47] FDA (US Food Drug Adm.). 2021. FDA recognition of public human genetic variant databases. Fact Sheet, FDA, Washington, DC. https://www.fda.gov/medical-devices/precision-medicine/fda-recognition-public-human-genetic-variant-databases

[R48] FDA (US Food Drug Adm.). 2022. FDA takes important steps to increase racial and ethnic diversity in clinical trials. Press Release, FDA, Washington, DC. https://www.fda.gov/news-events/press-announcements/fda-takes-important-steps-increase-racial-and-ethnic-diversity-clinical-trials

[R49] Federici G, Soddu S. 2020. Variants of uncertain significance in the era of high-throughput genome sequencing: a lesson from breast and ovary cancers. J. Exp. Clin. Cancer Res 39:46 32127026 10.1186/s13046-020-01554-6 PMC7055088

[R50] Feit NZ, Goldman DA, Smith E, Deighan J, Iasonos A, 2019. Use, safety, and efficacy of single-patient use of the US Food and Drug Administration Expanded Access Program. JAMA Oncol. 5:570–72 30816938 10.1001/jamaoncol.2018.7002 PMC6459119

[R51] Findlay GM, Boyle EA, Hause RJ, Klein JC, Shendure J. 2014. Saturation editing of genomic regions by multiplex homology-directed repair. Nature 513:120–23 25141179 10.1038/nature13695 PMC4156553

[R52] Flanagan SE, Patch AM, Ellard S. 2010. Using SIFT and PolyPhen to predict loss-of-function and gain-of-function mutations. Genet. Test Mol. Biomark 14:533–37 10.1089/gtmb.2010.0036 20642364

[R53] Fong PC, Boss DS, Yap TA, Tutt A, Wu P, 2009. Inhibition of poly(ADP-ribose) polymerase in tumors from *BRCA* mutation carriers. N. Engl. J. Med 361:123–34 19553641 10.1056/NEJMoa0900212

[R54] Fontana E, Valeri N. 2019. Class(y) dissection of *BRAF* heterogeneity: beyond non-V600. Clin. Cancer Res 25:6896–98 31585936 10.1158/1078-0432.CCR-19-2732

[R55] Friedman CF, Hainsworth JD, Kurzrock R, Spigel DR, Burris HA III, 2022. Atezolizumab treatment of tumors with high tumor mutational burden from MyPathway, a multicenter, open-label, phase IIa multiple basket study. Cancer Discov. 12:654–69 34876409 10.1158/2159-8290.CD-21-0450 PMC9394388

[R56] Ganesh K, Stadler ZK, Cercek A, Mendelsohn RB, Shia J, 2019. Immunotherapy in colorectal cancer: rationale, challenges and potential. Nat. Rev. Gastroenterol. Hepatol 16:361–75 30886395 10.1038/s41575-019-0126-x PMC7295073

[R57] Gasperini M, Starita L, Shendure J. 2016. The power of multiplexed functional analysis of genetic variants. Nat. Protoc 11:1782–87 27583640 10.1038/nprot.2016.135 PMC6690347

[R58] Gershenson DM, Miller A, Brady WE, Paul J, Carty K, 2022. Trametinib versus standard of care in patients with recurrent low-grade serous ovarian cancer (GOG 281/LOGS): an international, randomised, open-label, multicentre, phase 2/3 trial. Lancet 399:541–53 35123694 10.1016/S0140-6736(21)02175-9 PMC8819271

[R59] Goldberg KB, Blumenthal GM, McKee AE, Pazdur R. 2018. The FDA Oncology Center of Excellence and precision medicine. Exp. Biol. Med 243:308–12 10.1177/1535370217740861 PMC5813869 29105511

[R60] Gouda MA, Nelson BE, Buschhorn L, Wahida A, Subbiah V. 2023. Tumor-agnostic precision medicine from the AACR GENIE database: clinical implications. Clin. Cancer Res 29:2753–60 37061987 10.1158/1078-0432.CCR-23-0090 PMC10390861

[R61] Gouda MA, Subbiah V. 2023. Expanding the benefit: dabrafenib/trametinib as tissue-agnostic therapy for *BRAF* V600E–positive adult and pediatric solid tumors. Am. Soc. Clin. Oncol. Educ. Book 43:e404770 37159870 10.1200/EDBK_404770

[R62] Hainsworth JD, Meric-Bernstam F, Swanton C, Hurwitz H, Spigel DR, 2018. Targeted therapy for advanced solid tumors on the basis of molecular profiles: results from MyPathway, an open-label, phase IIa multiple basket study. J. Clin. Oncol 36:536–42 29320312 10.1200/JCO.2017.75.3780

[R63] Harada G, Yang SR, Cocco E, Drilon A. 2023. Rare molecular subtypes of lung cancer. Nat. Rev. Clin. Oncol 20:229–49 36806787 10.1038/s41571-023-00733-6 PMC10413877

[R64] Haslam A, Olivier T, Tuia J, Prasad V. 2023. A systematic review of basket and umbrella trials in oncology: the importance of tissue of origin and molecular target. Eur. J. Cancer 178:227–33 36493558 10.1016/j.ejca.2022.10.027

[R65] Heitzer E, Haque IS, Roberts CES, Speicher MR. 2019. Current and future perspectives of liquid biopsies in genomics-driven oncology. Nat. Rev. Genet 20:71–88 30410101 10.1038/s41576-018-0071-5

[R66] Hofmann MH, Gerlach D, Misale S, Petronczki M, Kraut N. 2022. Expanding the reach of precision oncology by drugging all *KRAS* mutants. Cancer Discov. 12:924–37 35046095 10.1158/2159-8290.CD-21-1331 PMC9394389

[R67] Hong DS, Fakih MG, Strickler JH, Desai J, Durm GA, 2020. KRAS^G12C^ inhibition with sotorasib in advanced solid tumors. N. Engl. J. Med 383:1207–17 32955176 10.1056/NEJMoa1917239 PMC7571518

[R68] Hyman DM, Piha-Paul SA, Won H, Rodon J, Saura C, 2018. HER kinase inhibition in patients with *HER2*- and *HER3*-mutant cancers. Nature 554:189–94 29420467 10.1038/nature25475 PMC5808581

[R69] Hyman DM, Puzanov I, Subbiah V, Faris JE, Chau I, 2015. Vemurafenib in multiple nonmelanoma cancers with *BRAF*^V600^ mutations. N. Engl. J. Med 373:726–36 26287849 10.1056/NEJMoa1502309 PMC4971773

[R70] Hyman DM, Smyth LM, Donoghue MTA, Westin SN, Bedard PL, 2017a. AKT inhibition in solid tumors with *AKT1* mutations. J. Clin. Oncol 35:2251–59 28489509 10.1200/JCO.2017.73.0143 PMC5501365

[R71] Hyman DM, Taylor BS, Baselga J. 2017b. Implementing genome-driven oncology. Cell 168:584–99 28187282 10.1016/j.cell.2016.12.015 PMC5463457

[R72] Iyer G, Deming DA, Demeure MJ, Federman N, McKean M, 2023. Phase 2, multicenter, open-label basket trial of nab-sirolimus for patients with malignant solid tumors harboring pathogenic inactivating alterations in *TSC1* or *TSC2* genes (PRECISION I). J. Clin. Oncol 41:TPS3168

[R73] Jensen K, Konnick EQ, Schweizer MT, Sokolova AO, Grivas P, 2021. Association of clonal hematopoiesis in DNA repair genes with prostate cancer plasma cell–free DNA testing interference. JAMA Oncol. 7:107–10 33151258 10.1001/jamaoncol.2020.5161 PMC7645740

[R74] Jhaveri K, Chang MT, Juric D, Saura C, Gambardella V, 2021. Phase I basket study of taselisib, an isoform-selective PI3K inhibitor, in patients with *PIK3CA*-mutant cancers. Clin. Cancer Res 27:447–59 33148674 10.1158/1078-0432.CCR-20-2657

[R75] Jonsson P, Bandlamudi C, Cheng ML, Srinivasan P, Chavan SS, 2019. Tumour lineage shapes *BRCA-*mediated phenotypes. Nature 571:576–79 31292550 10.1038/s41586-019-1382-1 PMC7048239

[R76] Kaizer AM, Koopmeiners JS, Kane MJ, Roychoudhury S, Hong DS, Hobbs BP. 2019. Basket designs: statistical considerations for oncology trials. JCO Precis. Oncol 3. 10.1200/PO.19.00194 PMC11637469 35100726

[R77] Kaluziak ST, Iafrate AJ, Karlovich CA, Williams PM, Sklar J, 2023. Discovery of gene fusions in driver-negative NCI-MATCH screening samples. J. Clin. Oncol 41:3112–12

[R78] Karlovich CA, Williams PM. 2019. Clinical applications of next-generation sequencing in precision oncology. Cancer J. 25:264–71 31335390 10.1097/PPO.0000000000000385 PMC6658137

[R79] Keller L, Belloum Y, Wikman H, Pantel K. 2021. Clinical relevance of blood-based ctDNA analysis: mutation detection and beyond. Br. J. Cancer 124:345–58 32968207 10.1038/s41416-020-01047-5 PMC7852556

[R80] Kemp SB, Cheng N, Markosyan N, Sor R, Kim IK, 2023. Efficacy of a small-molecule inhibitor of *Kras*^*G12D*^ in immunocompetent models of pancreatic cancer. Cancer Discov. 13:298–311 36472553 10.1158/2159-8290.CD-22-1066 PMC9900321

[R81] Kerr ID, Cox HC, Moyes K, Evans B, Burdett BC, 2017. Assessment of in silico protein sequence analysis in the clinical classification of variants in cancer risk genes. J. Community Genet 8:87–95 28050887 10.1007/s12687-016-0289-x PMC5386911

[R82] Kim D, Herdeis L, Rudolph D, Zhao Y, Böttcher J, 2023. Pan-KRAS inhibitor disables oncogenic signalling and tumour growth. Nature 619:160–66 37258666 10.1038/s41586-023-06123-3 PMC10322706

[R83] Klein H, Mazor T, Siegel E, Trukhanov P, Ovalle A, 2022. MatchMiner: an open-source platform for cancer precision medicine. npj Precis. Oncol 6:69 36202909 10.1038/s41698-022-00312-5 PMC9537311

[R84] Klute KA, Rothe M, Garrett-Mayer E, Mangat PK, Nazemzadeh R, 2022. Cobimetinib plus vemurafenib in patients with colorectal cancer with BRAF mutations: results from the Targeted Agent and Profiling Utilization Registry (TAPUR) study. JCO Precis. Oncol 6:e2200191 36409971 10.1200/PO.22.00191

[R85] Köhnke T, Majeti R. 2021. Clonal hematopoiesis: from mechanisms to clinical intervention. Cancer Discov. 11:2987–97 34407958 10.1158/2159-8290.CD-21-0901 PMC8854454

[R86] Kopetz S, Grothey A, Yaeger R, Van Cutsem E, Desai J, 2019. Encorafenib, binimetinib, and cetuximab in *BRAF* V600E–mutated colorectal cancer. N. Engl. J. Med 381:1632–43 31566309 10.1056/NEJMoa1908075

[R87] Krebs MG, Delord JP, Jeffry Evans TR, De Jonge M, Kim SW, 2023. Olaparib and durvalumab in patients with relapsed small cell lung cancer (MEDIOLA): an open-label, multicenter, phase 1/2, basket study. Lung Cancer 180:107216 37146473 10.1016/j.lungcan.2023.107216

[R88] Krzakowski MJ, Lu S, Cousin S, Smit EF, Springfeld C, 2022. Updated analysis of the efficacy and safety of entrectinib in patients (pts) with locally advanced/metastatic *NTRK* fusion–positive (NTRK-fp) solid tumors. J. Clin. Oncol 40(16 Suppl.):309935759726

[R89] Lai GGY, Lim TH, Lim J, Liew PJR, Kwang XL, 2019. Clonal *MET* amplification as a determinant of tyrosine kinase inhibitor resistance in epidermal growth factor receptor–mutant non-small-cell lung cancer. J. Clin. Oncol 37:876–84 30676858 10.1200/JCO.18.00177

[R90] Lara PN Jr., Paterniti DA, Chiechi C, Turrell C, Morain C, 2005. Evaluation of factors affecting awareness of and willingness to participate in cancer clinical trials. J. Clin. Oncol 23:9282–89 16361626 10.1200/JCO.2005.02.6245

[R91] Latham A, Srinivasan P, Kemel Y, Shia J, Bandlamudi C, 2019. Microsatellite instability is associated with the presence of Lynch syndrome pan-cancer. J. Clin. Oncol 37:286–95 30376427 10.1200/JCO.18.00283 PMC6553803

[R92] Lavacchi D, Roviello G, D’Angelo A. 2020. Tumor-agnostic treatment for cancer: when how is better than where. Clin. Drug Investig 40:519–27 10.1007/s40261-020-00915-5 32307639

[R93] Le DT, Durham JN, Smith KN, Wang H, Bartlett BR, 2017. Mismatch repair deficiency predicts response of solid tumors to PD-1 blockade. Science 357:409–13 28596308 10.1126/science.aan6733 PMC5576142

[R94] Le DT, Uram JN, Wang H, Bartlett BR, Kemberling H, 2015. PD-1 blockade in tumors with mismatch-repair deficiency. N. Engl. J. Med 372:2509–20 26028255 10.1056/NEJMoa1500596 PMC4481136

[R95] Lee JB, Jung M, Beom SH, Kim GM, Kim HR, 2021. Phase 2 study of TAS-117, an allosteric akt inhibitor in advanced solid tumors harboring phosphatidylinositol 3-kinase/v-akt murine thymoma viral oncogene homolog gene mutations. Investig. New Drugs 39:1366–74 33723724 10.1007/s10637-021-01085-7 PMC8426297

[R96] Lemery S, Keegan P, Pazdur R. 2017. First FDA approval agnostic of cancer site—when a biomarker defines the indication. N. Engl. J. Med 377:1409–12 29020592 10.1056/NEJMp1709968

[R97] Li BT, Daly B, Gospodarowicz M, Bertagnolli MM, Brawley OW, 2022a. Reimagining patient-centric cancer clinical trials: a multi-stakeholder international coalition. Nat. Med 28:620–26 35440725 10.1038/s41591-022-01775-6

[R98] Li BT, Shen R, Buonocore D, Olah ZT, Ni A, 2018. Ado-trastuzumab emtansine for patients with *HER2*-mutant lung cancers: results from a phase II basket trial. J. Clin. Oncol 36:2532–37 29989854 10.1200/JCO.2018.77.9777 PMC6366814

[R99] Li BT, Smit EF, Goto Y, Nakagawa K, Udagawa H, 2022b. Trastuzumab deruxtecan in *HER2*-mutant non-small-cell lung cancer. N. Engl. J. Med 386:241–51 34534430 10.1056/NEJMoa2112431 PMC9066448

[R100] Li MM, Cottrell CE, Pullambhatla M, Roy S, Temple-Smolkin RL, 2023. Assessments of somatic variant classification using the Association for Molecular Pathology/American Society of Clinical Oncology/College of American Pathologists guidelines: a report from the Association for Molecular Pathology. J. Mol. Diagn 25:69–86 36503149 10.1016/j.jmoldx.2022.11.002

[R101] Lim B, Lin Y, Navin N. 2020. Advancing cancer research and medicine with single-cell genomics. Cancer Cell 37:456–70 32289270 10.1016/j.ccell.2020.03.008 PMC7899145

[R102] Lito P, Solomon M, Li L-S, Hansen R, Rosen N. 2016. Allele-specific inhibitors inactivate mutant KRAS G12C by a trapping mechanism. Science 351:604–8 26841430 10.1126/science.aad6204 PMC4955282

[R103] Liu LY, Bhandari V, Salcedo A, Espiritu SMG, Morris QD, 2020. Quantifying the influence of mutation detection on tumour subclonal reconstruction. Nat. Commun 11:6247 33288765 10.1038/s41467-020-20055-w PMC7721877

[R104] Liu YL, Maio A, Kemel Y, Salo-Mullen EE, Sheehan M, 2022. Disparities in cancer genetics care by race/ethnicity among pan-cancer patients with pathogenic germline variants. Cancer 128:3870–79 36041233 10.1002/cncr.34434 PMC10335605

[R105] Lynam EB, Leaw J, Wiener MB. 2012. A patient focused solution for enrolling clinical trials in rare and selective cancer indications: a landscape of haystacks and needles. Drug Inf. J 46:472–78 23990689 10.1177/0092861512443436 PMC3754447

[R106] Mangat PK, Halabi S, Bruinooge SS, Garrett-Mayer E, Alva A, 2018. Rationale and design of the Targeted Agent and Profiling Utilization Registry (TAPUR) study. JCO Precis. Oncol 10.1200/PO.18.00122 PMC6312096 30603737

[R107] Mansfield AS, Wei Z, Mehra R, Shaw AT, Lieu CH, 2022. Crizotinib in patients with tumors harboring *ALK* or *ROS1* rearrangements in the NCI-MATCH trial. NPJ Precis. Oncol 6:13 35233056 10.1038/s41698-022-00256-w PMC8888601

[R108] Marabelle A, Fakih M, Lopez J, Shah M, Shapira-Frommer R, 2020a. Association of tumour mutational burden with outcomes in patients with advanced solid tumours treated with pembrolizumab: prospective biomarker analysis of the multicohort, open-label, phase 2 KEYNOTE-158 study. Lancet Oncol. 21:1353–65 32919526 10.1016/S1470-2045(20)30445-9

[R109] Marabelle A, Le DT, Ascierto PA, Di Giacomo AM, De Jesus-Acosta A, 2020b. Efficacy of pembrolizumab in patients with noncolorectal high microsatellite instability/mismatch repair-deficient cancer: results from the phase II KEYNOTE-158 Study. J. Clin. Oncol 38:1–10 31682550 10.1200/JCO.19.02105 PMC8184060

[R110] McCarthy AM, Bristol M, Domchek SM, Groeneveld PW, Kim Y, 2016. Health care segregation, physician recommendation, and racial disparities in *BRCA1*/*2* testing among women with breast cancer. J. Clin. Oncol 34:2610–18 27161971 10.1200/JCO.2015.66.0019 PMC5012689

[R111] Meric-Bernstam F, Bahleda R, Hierro C, Sanson M, Bridgewater J, 2022. Futibatinib, an irreversible FGFR1–4 inhibitor, in patients with advanced solid tumors harboring *FGF*/*FGFR* aberrations: a phase I dose-expansion study. Cancer Discov. 12:402–15 34551969 10.1158/2159-8290.CD-21-0697 PMC9762334

[R112] Meric-Bernstam F, Beeram M, Mayordomo JI, Hanna DL, Ajani JA, 2018. Single agent activity of ZW25, a HER2-targeted bispecific antibody, in heavily pretreated HER2-expressing cancers. J. Clin. Oncol 36:2500

[R113] Meric-Bernstam F, Hainsworth J, Bose R, Burris HA III, Friedman CF, 2021. MyPathway HER2 basket study: pertuzumab (P) + trastuzumab (H) treatment of a large, tissue-agnostic cohort of patients with *HER2*-positive advanced solid tumors. J. Clin. Oncol 39:3004 10.1200/JCO.22.02636 PMC10824375 37793085

[R114] Meric-Bernstam F, Hurwitz H, Raghav KPS, McWilliams RR, Fakih M, 2019. Pertuzumab plus trastuzumab for *HER2*-amplified metastatic colorectal cancer (MyPathway): an updated report from a multicentre, open-label, phase 2a, multiple basket study. Lancet Oncol. 20:518–30 30857956 10.1016/S1470-2045(18)30904-5 PMC6781620

[R115] Meric-Bernstam F, Makker V, Oaknin A, Oh D-Y, Banerjee SN, 2023. Efficacy and safety of trastuzumab deruxtecan (T-DXd) in patients (pts) with HER2-expressing solid tumors: DESTINY-PanTumor02 (DP-02) interim results. J. Clin. Oncol 41:LBA3000 10.1200/JCO.23.02005 PMC10730032 37870536

[R116] Mohd Noor A, Sarker D, Vizor S, McLennan B, Hunter S, 2013. Effect of patient socioeconomic status on access to early-phase cancer trials. J. Clin. Oncol 31:224–30 23213088 10.1200/JCO.2012.45.0999

[R117] Moore DC, Guinigundo AS. 2023. The role of biomarkers in guiding clinical decision-making in oncology. J. Adv. Pract. Oncol 14:15–37 37206905 10.6004/jadpro.2023.14.3.17 PMC10190804

[R118] Morganti S, Tarantino P, Ferraro E, D’Amico P, Viale G, 2019. Complexity of genome sequencing and reporting: next generation sequencing (NGS) technologies and implementation of precision medicine in real life. Crit. Rev. Oncol. Hematol 133:171–82 30661654 10.1016/j.critrevonc.2018.11.008

[R119] Murciano-Goroff YR, Drilon A, Stadler ZK. 2021. The NCI-MATCH: a national, collaborative precision oncology trial for diverse tumor histologies. Cancer Cell 39:22–24 33434511 10.1016/j.ccell.2020.12.021 PMC10640715

[R120] Murciano-Goroff YR, Schram AM, Rosen EY, Won H, Gong Y, 2022. Reversion mutations in germline *BRCA1*/*2*-mutant tumors reveal a BRCA-mediated phenotype in non-canonical histologies. Nat. Commun 13:7182 36418296 10.1038/s41467-022-34109-8 PMC9684575

[R121] Murciano-Goroff YR, Taylor BS, Hyman DM, Schram AM. 2020a. Toward a more precise future for oncology. Cancer Cell 37:431–42 32289268 10.1016/j.ccell.2020.03.014 PMC7499397

[R122] Murciano-Goroff YR, Warner AB, Wolchok JD. 2020b. The future of cancer immunotherapy: microenvironment-targeting combinations. Cell Res. 30:507–19 32467593 10.1038/s41422-020-0337-2 PMC7264181

[R123] Murthy VH, Krumholz HM, Gross CP. 2004. Participation in cancer clinical trials: race-, sex-, and age-based disparities. JAMA 291:2720–26 15187053 10.1001/jama.291.22.2720

[R124] Nagasaka M, Ou S-HI. 2022. *NRG1* and *NRG2* fusion positive solid tumor malignancies: a paradigm of ligand-fusion oncogenesis. Trends Cancer 8:242–58 34996744 10.1016/j.trecan.2021.11.003

[R125] Oleary S, Shulman M, Ritt K, Degele M, Protomastro E, 2021. The TIME Trial Network to facilitate rapid clinical trial activation, patient screening, and enrollment in molecularly targeted trials. J. Clin. Oncol 39:1563–6333822655

[R126] Ou S-HI, Jänne PA, Leal TA, Rybkin II, Sabari JK, 2022. First-in-human phase I/Ib dose-finding study of adagrasib (MRTX849) in patients with advanced *KRAS*^*G12C*^ solid tumors (KRYSTAL-1). J. Clin. Oncol 40:2530–38 35167329 10.1200/JCO.21.02752 PMC9362872

[R127] Patel SP, Othus M, Chae YK, Giles FJ, Hansel DE, 2020. A phase II basket trial of dual anti-CTLA-4 and anti-PD-1 blockade in rare tumors (DART SWOG 1609) in patients with nonpancreatic neuroendocrine tumors. Clin. Cancer Res 26:2290–96 31969335 10.1158/1078-0432.CCR-19-3356 PMC7231627

[R128] Perez CA, Henry JT, Varkaris A, Subbiah V, Spira AI, 2022. First-in-human global multi-center study of RLY-2608, a pan-mutant and isoform-selective PI3Kα inhibitor, as a single agent in patients with advanced solid tumors and in combination with fulvestrant in patients with advanced breast cancer. J. Clin. Oncol 40:TPS1124

[R129] Polak TB, Cucchi DGJ, Schelhaas J, Ahmed SS, Khoshnaw N, 2023. Results from expanded access programs: a review of academic literature. Drugs 83:795–805 37199856 10.1007/s40265-023-01879-4 PMC10193319

[R130] Prahallad A, Sun C, Huang S, Di Nicolantonio F, Salazar R, 2012. Unresponsiveness of colon cancer to BRAF(V600E) inhibition through feedback activation of EGFR. Nature 483:100–3 22281684 10.1038/nature10868

[R131] Ptashkin RN, Mandelker DL, Coombs CC, Bolton K, Yelskaya Z, 2018. Prevalence of clonal hematopoiesis mutations in tumor-only clinical genomic profiling of solid tumors. JAMA Oncol. 4:1589–93 29872864 10.1001/jamaoncol.2018.2297 PMC6224316

[R132] Rodon J, Damian S, Furqan M, Garcia-Donas J, Imai H, 2023. Clinical and translational findings of pemigatinib in previously treated solid tumors with activating *FGFR1–3* alterations in the FIGHT-207 study. Cancer Res. 83:CT016 (abstr.)

[R133] Rosen E, Drilon A, Chakravarty D. 2022. Precision oncology: 2022 in review. Cancer Discov. 12:2747–53 36458431 10.1158/2159-8290.CD-22-1154

[R134] Salama AKS, Li S, Macrae ER, Park JI, Mitchell EP, 2020. Dabrafenib and trametinib in patients with tumors with *BRAF*^*V600E*^ mutations: results of the NCI-MATCH trial subprotocol H. J. Clin. Oncol 38:3895–904 32758030 10.1200/JCO.20.00762 PMC7676884

[R135] Samstein RM, Lee CH, Shoushtari AN, Hellmann MD, Shen R, 2019. Tumor mutational load predicts survival after immunotherapy across multiple cancer types. Nat. Genet 51:202–6 30643254 10.1038/s41588-018-0312-8 PMC6365097

[R136] Scepura B, Chan M, Kim T, Boehmer J, Goldberg KB, Pazdur R. 2021. Oncology expanded access and FDA’s Project Facilitate. Oncologist 26:e1880–82 34288259 10.1002/onco.13910 PMC8488788

[R137] Schram AM, Chang MT, Jonsson P, Drilon A. 2017. Fusions in solid tumours: diagnostic strategies, targeted therapy, and acquired resistance. Nat. Rev. Clin. Oncol 14:735–48 28857077 10.1038/nrclinonc.2017.127 PMC10452928

[R138] Schram AM, Colombo N, Arrowsmith E, Narayan V, Yonemori K, 2023a. Avelumab plus talazoparib in patients with *BRCA1*/*2*- or *ATM*-altered advanced solid tumors: results from JAVELIN BRCA/ATM, an open-label, multicenter, phase 2b, tumor-agnostic trial. JAMA Oncol. 9:29–39 36394867 10.1001/jamaoncol.2022.5218 PMC9673021

[R139] Schram AM, Goto K, Kim D-W, Martin-Romano P, Ou S-HI, 2022. Efficacy and safety of zenocutuzumab, a *HER2* × *HER3* bispecific antibody, across advanced *NRG1* fusion (*NRG1*^+^) cancers. J. Clin. Oncol 40:10534652953

[R140] Schram AM, Subbiah V, Sullivan RJ, Cosman R, Liu J, 2023b. A first-in-human, phase 1a/1b, open-label, dose-escalation and expansion study to investigate the safety, pharmacokinetics, and antitumor activity of the RAF dimer inhibitor BGB-3245 in patients with advanced or refractory tumors. Cancer Res. 83:CT031 (abstr.)

[R141] Serrano MJ, Garrido-Navas MC, Diaz Mochon JJ, Cristofanilli M, Gil-Bazo I, 2020. Precision prevention and cancer interception: the new challenges of liquid biopsy. Cancer Discov. 10:1635–44 33037026 10.1158/2159-8290.CD-20-0466

[R142] Sharrocks K, Spicer J, Camidge DR, Papa S. 2014. The impact of socioeconomic status on access to cancer clinical trials. Br. J. Cancer 111:1684–87 25093493 10.1038/bjc.2014.108 PMC4453719

[R143] Shrestha Bhattarai T, Shamu T, Gorelick AN, Chang MT, Chakravarty D, 2022. *AKT* mutant allele–specific activation dictates pharmacologic sensitivities. Nat. Commun 13:2111 35440569 10.1038/s41467-022-29638-1 PMC9018718

[R144] Simon R, Roychowdhury S. 2013. Implementing personalized cancer genomics in clinical trials. Nat. Rev. Drug Discov 12:358–69 23629504 10.1038/nrd3979

[R145] Siozopoulou V, Smits E, De Winne K, Marcq E, Pauwels P. 2021. *NTRK* fusions in sarcomas: diagnostic challenges and clinical aspects. Diagnostics 11:478 33803146 10.3390/diagnostics11030478 PMC8000177

[R146] Sivakumar S, Jin DX, Rathod R, Ross J, Cantley LC, 2023. Genetic heterogeneity and tissue-specific patterns of tumors with multiple *PIK3CA* mutations. Clin. Cancer Res 29:1125–36 36595567 10.1158/1078-0432.CCR-22-2270 PMC10011881

[R147] Skoulidis F, Li BT, Dy GK, Price TJ, Falchook GS, 2021. Sotorasib for lung cancers with *KRAS* p.G12C mutation. N. Engl. J. Med 384:2371–81 34096690 10.1056/NEJMoa2103695 PMC9116274

[R148] Subbiah V, Kreitman RJ, Wainberg ZA, Cho JY, Schellens JHM, 2022a. Dabrafenib plus trametinib in patients with *BRAF* V600E–mutant anaplastic thyroid cancer: updated analysis from the phase II ROAR basket study. Ann. Oncol 33:406–15 35026411 10.1016/j.annonc.2021.12.014 PMC9338780

[R149] Subbiah V, Kreitman RJ, Wainberg ZA, Gazzah A, Lassen U, 2023a. Dabrafenib plus trametinib in *BRAF*^*V600E*^-mutated rare cancers: the phase 2 ROAR trial. Nat. Med 29:1103–12 37059834 10.1038/s41591-023-02321-8 PMC10202803

[R150] Subbiah V, Lassen U, Élez E, Italiano A, Curigliano G, 2020. Dabrafenib plus trametinib in patients with *BRAF*^V600E^-mutated biliary tract cancer (ROAR): a phase 2, open-label, single-arm, multicentre basket trial. Lancet Oncol. 21:1234–43 32818466 10.1016/S1470-2045(20)30321-1

[R151] Subbiah V, Sahai V, Maglic D, Bruderek K, Toure BB, 2023b. RLY-4008, the first highly selective FGFR2 inhibitor with activity across *FGFR2* alterations and resistance mutations. Cancer Discov. 13:2012–31 37270847 10.1158/2159-8290.CD-23-0475 PMC10481131

[R152] Subbiah V, Wolf J, Konda B, Kang H, Spira A, 2022b. Tumour-agnostic efficacy and safety of selpercatinib in patients with RET fusion–positive solid tumours other than lung or thyroid tumours (LIBRETTO-001): a phase 1/2, open-label, basket trial. Lancet Oncol. 23:1261–73 36108661 10.1016/S1470-2045(22)00541-1 PMC11702314

[R153] Sullivan RJ, Hollebecque A, Flaherty KT, Shapiro GI, Rodon Ahnert J, 2020. A phase I study of LY3009120, a pan-RAF inhibitor, in patients with advanced or metastatic cancer. Mol. Cancer Ther 19:460–67 31645440 10.1158/1535-7163.MCT-19-0681

[R154] Sullivan RJ, Infante JR, Janku F, Wong DJL, Sosman JA, 2018. First-in-class ERK1/2 inhibitor ulixertinib (BVD-523) in patients with *MAPK* mutant advanced solid tumors: results of a phase I dose-escalation and expansion study. Cancer Discov. 8:184–95 29247021 10.1158/2159-8290.CD-17-1119

[R155] Syn NL, Yong WP, Goh BC, Lee SC. 2016. Evolving landscape of tumor molecular profiling for personalized cancer therapy: a comprehensive review. Expert Opin. Drug Metab. Toxicol 12:911–22 27249175 10.1080/17425255.2016.1196187

[R156] Tanaka H, Watanabe T. 2020. Mechanisms underlying recurrent genomic amplification in human cancers. Trends Cancer 6:462–77 32383436 10.1016/j.trecan.2020.02.019 PMC7285850

[R157] Tao JJ, Eubank MH, Schram AM, Cangemi N, Pamer E, 2019. Real-world outcomes of an automated physician support system for genome-driven oncology. JCO Precis. Oncol 3:PO.19.000066 10.1200/PO.19.00066 PMC7446398 32914018

[R158] Tao JJ, Schram AM, Hyman DM. 2018. Basket studies: redefining clinical trials in the era of genome-driven oncology. Annu. Rev. Med 69:319–31 29120700 10.1146/annurev-med-062016-050343 PMC7455011

[R159] Tateo V, Marchese PV, Mollica V, Massari F, Kurzrock R, Adashek JJ. 2023. Agnostic approvals in oncology: getting the right drug to the right patient with the right genomics. Pharmaceuticals 16:614 37111371 10.3390/ph16040614 PMC10144220

[R160] Tejeda HA, Green SB, Trimble EL, Ford L, High JL, 1996. Representation of African-Americans, Hispanics, and whites in National Cancer Institute cancer treatment trials. J. Natl. Cancer Inst 88:812–16 8637047 10.1093/jnci/88.12.812

[R161] Torkamani A, Schork NJ. 2008. Prediction of cancer driver mutations in protein kinases. Cancer Res. 68:1675–82 18339846 10.1158/0008-5472.CAN-07-5283

[R162] Tsimberidou AM, Fountzilas E, Nikanjam M, Kurzrock R. 2020. Review of precision cancer medicine: evolution of the treatment paradigm. Cancer Treat. Rev 86:102019 32251926 10.1016/j.ctrv.2020.102019 PMC7272286

[R163] Uehara Y, Koyama T, Katsuya Y, Sato J, Yamamoto N. 2023. Impact of patient travel time on disparities in precision oncology clinical trials. J. Clin. Oncol 41:3113–13 10.1016/j.esmorw.2025.100114 PMC12836732 41647335

[R164] Unger JM, Moseley AB, Cheung CK, Osarogiagbon RU, Symington B, 2021. Persistent disparity: socioeconomic deprivation and cancer outcomes in patients treated in clinical trials. J. Clin. Oncol 39:1339–48 33729825 10.1200/JCO.20.02602 PMC8078474

[R165] Unni AM, Lockwood WW, Zejnullahu K, Lee-Lin SQ, Varmus H. 2015. Evidence that synthetic lethality underlies the mutual exclusivity of oncogenic *KRAS* and *EGFR* mutations in lung adenocarcinoma. eLife 4:e06907 26047463 10.7554/eLife.06907 PMC4478584

[R166] US Dep. Health Hum. Serv., FDA (US Food Drug Adm.), OCE (Oncol. Cent. Excel.), CDER (Cent. Drug Eval. Res.), CBER (Cent. Biol. Eval. Res.). 2022. Tissue agnostic drug development in oncology: guidance for industry. Draft Guid., FDA, Washington, DC. https://www.fda.gov/media/162346/download

[R167] Vasan N, Baselga J, Hyman DM. 2019. A view on drug resistance in cancer. Nature 575:299–309 31723286 10.1038/s41586-019-1730-1 PMC8008476

[R168] Wagner AH, Walsh B, Mayfield G, Tamborero D, Sonkin D, 2020. A harmonized meta-knowledgebase of clinical interpretations of somatic genomic variants in cancer. Nat. Genet 52:448–57 32246132 10.1038/s41588-020-0603-8 PMC7127986

[R169] Wahida A, Buschhorn L, Fröhling S, Jost PJ, Schneeweiss A, 2023. The coming decade in precision oncology: six riddles. Nat. Rev. Cancer 23:43–54 36434139 10.1038/s41568-022-00529-3

[R170] Wang TS, Lee C, Severson P, Pelham RJ, Williams R, Miller NLG. 2023. Exarafenib (KIN-2787) is a potent, selective pan-RAF inhibitor with activity in preclinical models of *BRAF* class II/III mutant and *NRAS* mutant melanoma. Cancer Res. 83:4927 (abstr.)

[R171] Wen PY, Stein A, van den Bent M, De Greve J, Wick A, 2022. Dabrafenib plus trametinib in patients with *BRAF*^V600E^-mutant low-grade and high-grade glioma (ROAR): a multicentre, open-label, single-arm, phase 2, basket trial. Lancet Oncol. 23:53–64 34838156 10.1016/S1470-2045(21)00578-7

[R172] West HJ. 2017. Novel precision medicine trial designs: umbrellas and baskets. JAMA Oncol. 3:423–23 27930754 10.1001/jamaoncol.2016.5299

[R173] Wiener MB, Newman HM, Spradley EA. 2007. Revolutionizing oncology patient enrollment in clinical trials: just-in-time approach. J. Clin. Oncol 25:6577–77

[R174] Wirth LJ, Sherman E, Robinson B, Solomon B, Kang H, 2020. Efficacy of selpercatinib in *RET*-altered thyroid cancers. N. Engl. J. Med 383:825–35 32846061 10.1056/NEJMoa2005651 PMC10777663

[R175] Wisinski KB, Flamand Y, Wilson MA, Luke JJ, Tawbi HA, 2023. Trametinib in patients with *NF1*-, *GNAQ*-, or *GNA11*-mutant tumors: results from the NCI-MATCH ECOG-ACRIN trial (EAY131) subprotocols S1 and S2. JCO Precis. Oncol 7:e2200421 37053535 10.1200/PO.22.00421 PMC10309549

[R176] Yaeger R, Weiss J, Pelster MS, Spira AI, Barve M, 2023. Adagrasib with or without cetuximab in colorectal cancer with mutated *KRAS G12C*. N. Engl. J. Med 388:44–54 36546659 10.1056/NEJMoa2212419 PMC9908297

[R177] Yao Z, Gao Y, Su W, Yaeger R, Tao J, 2019. RAF inhibitor PLX8394 selectively disrupts BRAF dimers and RAS-independent BRAF-mutant-driven signaling. Nat. Med 25:284–91 30559419 10.1038/s41591-018-0274-5 PMC6404779

[R178] Yao Z, Torres NM, Tao A, Gao Y, Luo L, 2015. *BRAF* mutants evade ERK-dependent feedback by different mechanisms that determine their sensitivity to pharmacologic inhibition. Cancer Cell 28:370–83 26343582 10.1016/j.ccell.2015.08.001 PMC4894664

[R179] Yao Z, Yaeger R, Rodrik-Outmezguine VS, Tao A, Torres NM, 2017. Tumours with class 3 BRAF mutants are sensitive to the inhibition of activated RAS. Nature 548:234–38 28783719 10.1038/nature23291 PMC5648058

[R180] Yap TA, Tan DSP, Terbuch A, Caldwell R, Guo C, 2021. First-in-human trial of the oral ataxia telangiectasia and RAD3-related (ATR) inhibitor BAY 1895344 in patients with advanced solid tumors. Cancer Discov. 11:80–91 32988960 10.1158/2159-8290.CD-20-0868 PMC9554790

[R181] Zhou H, Liu F, Wu C, Rubin EH, Giranda VL, Chen C. 2019. Optimal two-stage designs for exploratory basket trials. Contemp. Clin. Trials 85:105807 31260789 10.1016/j.cct.2019.06.021

